# Recent Advances of Biochar-Based Electrochemical Sensors and Biosensors

**DOI:** 10.3390/bios12060377

**Published:** 2022-05-31

**Authors:** Yunxiao Li, Rui Xu, Huabin Wang, Wumei Xu, Liyan Tian, Jingxin Huang, Chengyue Liang, Yong Zhang

**Affiliations:** Provincial Key Laboratory of Rural Energy Engineering in Yunnan, Yunnan Normal University, Kunming 650500, China; ynnu_liyx@126.com (Y.L.); hbwang@ynnu.edu.cn (H.W.); xuwumei@ynnu.edu.cn (W.X.); tianly@ynnu.edu.cn (L.T.); huangjingxin17@163.com (J.H.); liangcy@ynnu.edu.cn (C.L.)

**Keywords:** biochar, electrochemical sensor, electrochemical biosensor, heavy metal, environmental pollutant

## Abstract

In the context of accelerating the global realization of carbon peaking and carbon neutralization, biochar produced from biomass feedstock via a pyrolysis process has been more and more focused on by people from various fields. Biochar is a carbon-rich material with good properties that could be used as a carrier, a catalyst, and an absorbent. Such properties have made biochar a good candidate as a base material in the fabrication of electrochemical sensors or biosensors, like carbon nanotube and graphene. However, the study of the applications of biochar in electrochemical sensing technology is just beginning; there are still many challenges to be conquered. In order to better carry out this research, we reviewed almost all of the recent papers published in the past 5 years on biochar-based electrochemical sensors and biosensors. This review is different from the previously published review papers, in which the types of biomass feedstock, the preparation methods, and the characteristics of biochar were mainly discussed. First, the role of biochar in the fabrication of electrochemical sensors and biosensors is summarized. Then, the analytes determined by means of biochar-based electrochemical sensors and biosensors are discussed. Finally, the perspectives and challenges in applying biochar in electrochemical sensors and biosensors are provided.

## 1. Introduction

As one of modern analytical techniques, electrochemical sensing technology is focused on by people from various fields that include food analysis, environmental monitoring, clinic diagnosis, and agricultural detection [1]. As we all know, with the development of nanotechnology, micro-nanomaterials-based electrochemical sensors and biosensors have shown increasingly excellent analytical performance in terms of sensitivity, linear range, limit of detection, testing time, stability, selectivity, and repeatability [2]. Among the advanced functional micro-nanomaterials, carbon-based materials have always played an important role in fabricating electrochemical sensing platforms. One of the most familiar materials is graphene. Since the use of graphene was developed, it has been sought after by researchers in various fields because of its perfect characteristics. In electrochemical-sensing studies, graphene can be used as a carrier, a catalyst, or a conductive basement, as well as framework material. Recently, as a member of the carbon family, biochar has been increasingly of interest to researchers. The reasons include its cost-effectiveness and its eco-friendly materials, as well as its comparison with graphene and carbon nanotubes.

Biochar is a carbon-rich material produced from biomass feedstock via a pyrolysis process under an anaerobic or anoxic condition [3,4,5]. Most of its raw materials are obtained from agricultural tailing and forestry ecosystem wastes (such as plant and animal residues) or municipal wastes (such as dewatered sludge); therefore, biochar is regarded as a product of biomass feedstock utilization [6,7,8]. With the growth of green chemistry concepts, the preparation and application of biochar have been receiving increased attention [9]. Song et al., reviewed the evolution of biochar research from its initial budding to its diversified development between 1999 and 2021 and showed that the study trend of biochar had increased gradually from 2014; those studies made outstanding contributions to the scientific research on energy and environment [6]. The main reason for the focus on biochar is that it presents amorphous characteristics, a large specific surface area, and good stability, characteristics that are similar to those of carbon nanotube and graphene structures. Furthermore, the synthesis of biochar may be carried out at different pyrolysis temperatures, depending on the type of polarity (hydrophilic or hydrophobic) that is desired, in situations where hydrophilic polarity is regarded as an important feature for ensuring good homogeneity of the material in an aqueous medium [10]. In addition to biochar’s properties, it is worth noting that biochar is ubiquitous, versatile, and inexpensive [11]. All of these advantages of biochar provide it with the potential to be utilized for electrode construction and fabricating cost-effective sensors.

The study of biochar on a large scale started in 2010 and moved to a reactivity exploration stage from 2014; the first works were reported in 1999 [6]. In order to better summarize the existing research results and to carry out more in-depth research on biochar, many review papers were published in the past decade [12,13,14,15,16]. Some comprehensive reviews identified biochar as a functional and novel material, in the context of a general discussion. For instance, Sharma et al., summarized the use of porous nanomaterials in the application of agricultural nanotechnology [17]. Torrinha et al., summarized the nanostructured carbon materials in the application of electrochemical sensors and biosensors for the screening of emerging pharmaceutical pollutants in waters and aquatic species [18]. Arduini et al., reviewed sustainable materials for the design of forefront printed (bio) sensors applied in the agrifood sector [19].

Recently, several review papers have been published to introduce the application of biochar in the sensor research field [20,21,22,23]. For example, Kalinke et al., addressed the use of biochar for the development of electrochemical devices with a focus on three aspects: the history of biochar, the technologies for improving biochar’s properties, and the electrochemical applications of biochar in the fields of sensors and biosensors, energy storage, and production [21]. Nevertheless, few review articles have addressed biochar-based electrochemical sensors and biosensors focusing on analytes, or biochar’s role in the fabrication process.

**Scheme 1:**
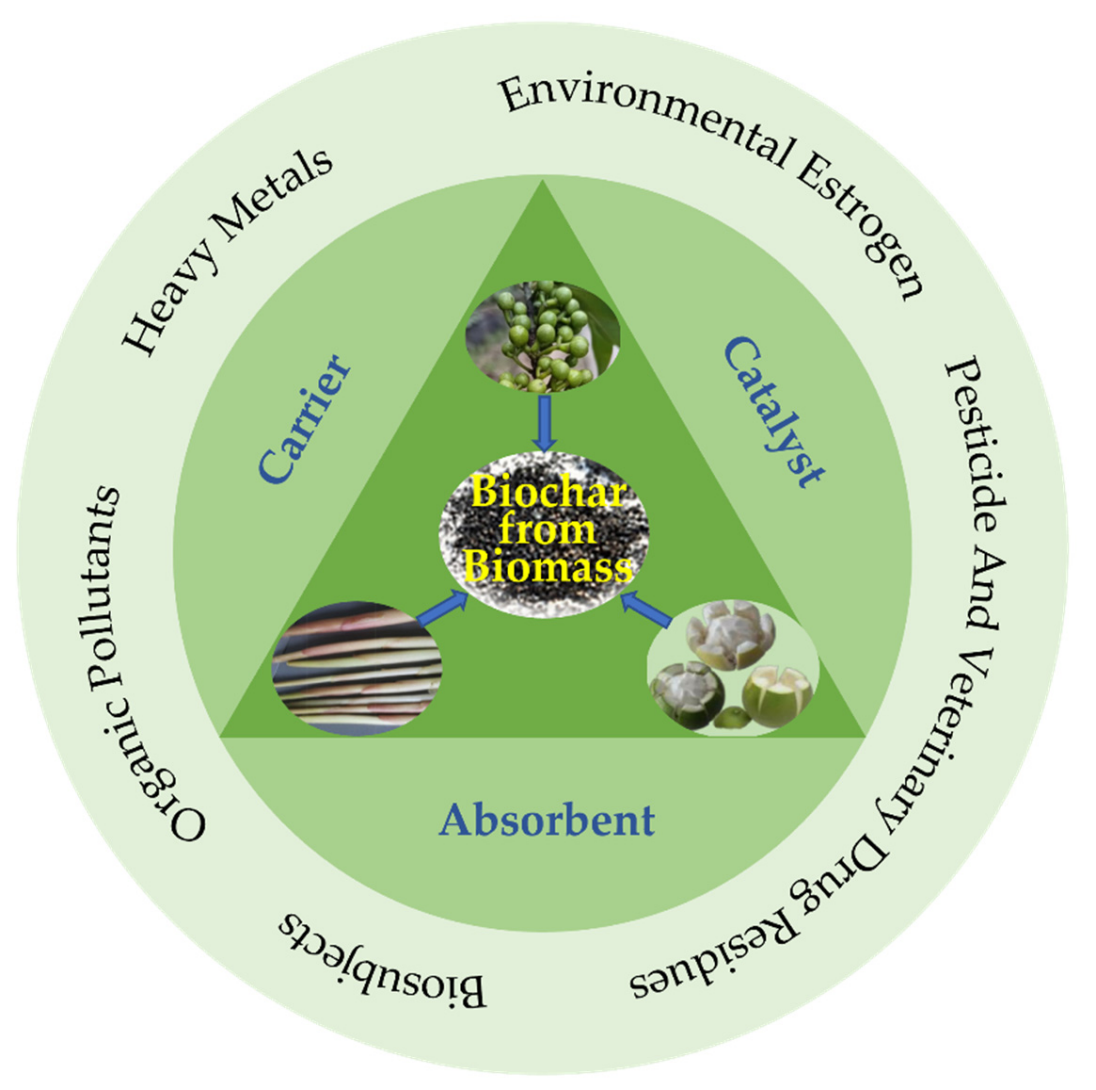
Schematics illustrating the application of biochar-based electrochemical sensors and biosensors, as reviewed in this paper.

In this review, we summarized almost all recent papers, in the past 5 years, on biochar-based electrochemical sensors and biosensors. This review is different from previously published review papers, in which the types of biomass feedstock, the preparation methods, and the characteristics of biochar were mainly discussed. As shown in Scheme 1, the role of biochar in the fabrication of electrochemical sensors and biosensors will first be summarized in this review. Then, the analytes (i.e., the detection target) determined by means of biochar-based electrochemical sensors and biosensors will be discussed. Finally, the perspectives and challenges in applying biochar in electrochemical sensors and biosensors will be provided.

## 2. The Role of Biochar in the Fabrication of Electrochemical Sensors and Biosensors

In the last three decades, the studies of electrochemical sensors and biosensors have made great progress, with the development of nanomaterials and nanotechnologies. As the research and the applications of electrochemical sensors and biosensors continue to develop, a single-handed pursuit of accuracy and sensitivity cannot meet the demands of the analysis in many in situ or point-of-care testing (POCT) circumstances, especially in the fields of food analysis, clinical diagnosis, environmental monitoring, and agricultural detection [24]. More cost-effective, stable, and versatile electrodes, as well as more stable and repeatable sensing strategies, are needed. Generally, electrochemical (bio) sensing technology relies on modified electrode surfaces for the transduction of redox reactions. The signal generated by the transfer of electrons between the transducer and the analyte is amplified in the equipment and finally displayed [18]. To realize that purpose, biochar has emerged in the attention of researchers. In addition to its advantages mentioned above (i.e., amorphous characteristics, a large specific surface area, and good stability, etc.), biochar has highly reactive and surface-functionalized spherical and porous structures [20]. Specifically, it presents negatively or positively charged sites, which allow for the easy electrostatic attraction (or repulsion) of charged compounds [21]. By virtue of these advantages, biochar is a good candidate material for electrode fabrication or modification, and it has appeared in various roles in preparing electrochemical sensors and biosensors.

### 2.1. Absorbent

The highly porous structure of biochar makes it ideal for use as an absorbent [14,16]. The detection of toxic heavy metals is always accompanied by a procedure of absorption. Accordingly, the detection process is generally carried out via two steps. The first step is the absorption and accumulation of heavy metals; the second step is electrochemical measurement. For instance, Liu et al. prepared water-processable biochar from pyrolysis of pitch pine to modify a glass carbon electrode [25]. The prepared electrode was immersed into a stirring 0.1 M acetate buffer solution (pH 5.0) containing Cd^2+^ to absorb and accumulate Cd^2+^ on the surface of the electrode. Following that, they used square wave anodic stripping voltammetry (SWASV) under optimized conditions to detect the Cd^2+^ in a linear range of 2.0 × 10^−8^ to 8 × 10^−5^ M, with a limit of detection (LOD) of 6.7 nM. 

**Figure 1:**
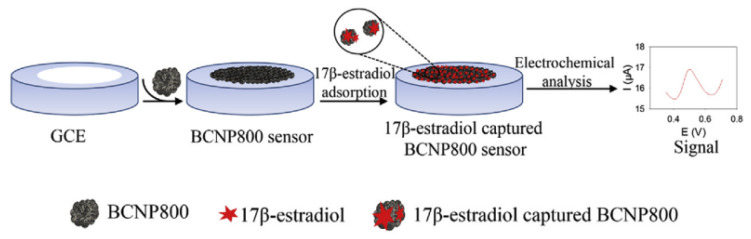
Schematics for the illustration of the procedures for the biochar-based electrochemical sensor construction and the electrochemical detection of 17*β*-estradiol. Reprinted with permission from Ref. [27]. Copyright © 2018, Elsevier.

The functionalization of renewable carbon could result in an increase of carboxyl, nitro, carbonyl, and sulfur-based groups on the surface of biochar. The great adsorption capacity of biochar is not applicable to heavy metal ions, but also to organic molecules, such as escitalopram [26]. Therefore, this capacity provides for the possibility of electrochemical detection of organic pollutants by means of biocharmodified electrodes. As shown in Figure 1, Dong et al., developed a sensitive and cost-effective electrochemical sensor based on highly conductive and adsorptive biochar nanoparticles (BCNPs) for the electrochemical detection of 17β-estradiol [27]. They investigated the electrochemical properties of the BCNPs with variations in the pyrolysis conditions and found that BCNPs obtained at the pyrolysis temperature of 800 °C (named BCNP800) exhibited high conductivity. Utilizing BCNP800 for absorbing 17β-estradiol on the surface of the electrode, the fabricated electrochemical sensor obtained improved amperometric responses, such as lowered anodic potential, decreased impedance, and an enhanced current signal toward 17β-estradiol, compared with the responses obtained via a pristine glass carbon electrode (GCE). With the synergetic effect of the high adsorption and conductivity properties of the BCNP800 sensor, an LOD of 11.30 nM was obtained.

Although the porous structure of biochar causes it to have great absorptive capability, the natural frame structure of biomass is often damaged or collapses in the process of preparing biochar, which is not conductive. This is also a great limitation in applying biochar in the electrochemical studies. To make it conductive, and thus to make it useful in electrochemical detection, biochar must always be used after absorbing conductive materials or redox metals. For example, Cu^2+^/Cu^+^ is a good pair of redox metals ions that shows excellent electrical conductivity in speeding up the charge transfer rate. Recently, several electrochemical sensors based on Cu^2+^/Cu^+^-biochar composites were prepared [28,29,30]. In contrast with other preparation of biochar by high temperature pyrolysis, the preparation procedure initially mixes the Cu source with biomass feedstock. Then, the Cu^2+^/Cu^+^-biochar composites are obtained through the pyrolysis of the mixture at 250 °C. Due to the unique 3D porous network structure of biochar, which increases the contact area and the absorption to targets, and to the excellent electrical conductivity of the Cu^2+^/Cu^+^ ions pair that speeds up the charge transfer rate, the fabricated electrochemical sensor shows good performance in accumulation and electrochemical response.

### 2.2. Catalyst

It is worth noting that biochar can also perform as a catalyst, as well as exhibiting its porous properties. For example, Oliveira et al., first prepared biochar from castor oil cake biomass, and then activated it by chemical activation treatment to increase the amount of surface functional groups and thus to improve the preconcentration capacity of the methyl parathion [31]. As the preconcentration step was spontaneous, without electric potential application, the assay interference of concomitant species could be effectively minimized. Under the potential of ~−0.5 V, the activated biochar exhibited good electrochemical reduction of methyl parathion. The sensitivity and LOD of the sensor were 760 μA·mM and 39.0 nM, respectively.

As stated in the published papers, together with its inherent strong adsorption property, biochar’s good catalytic property causes it to have even more potential in the fabrication of electrochemical sensors and biosensors. However, the catalytic mechanism by means of biochar used directly in the electrochemical reaction still needs to be explored more thoroughly. The effect of biochar’s morphology might be one of the key factors for the enhancement of the catalytic ability of biochar. For example, Bukhari et al., prepared a type of water-dispersed biochar, with a nanofiber structure, from pyrolysis of eucalyptus scraps [32]. The prepared biochar-nanofibers (BH-CNF) exhibited good electrochemical catalytic capacities for phenolic compounds. Based on the different potentials of different phenolic compounds obtained from the BH-CNF modified screen-printed electrodes, they developed several electrochemical sensors for sensing phenolic compound couples as follows: dopamine/L-tyrosine as phenols containing an amino group, caffeic acid/*p*-coumaric acid as hydroxycinnamic acids, hydroxytyrosol/tyrosol as a representative of phenylethanoids, and catechol/*m*-cresol as simple phenols.

**Figure 2:**
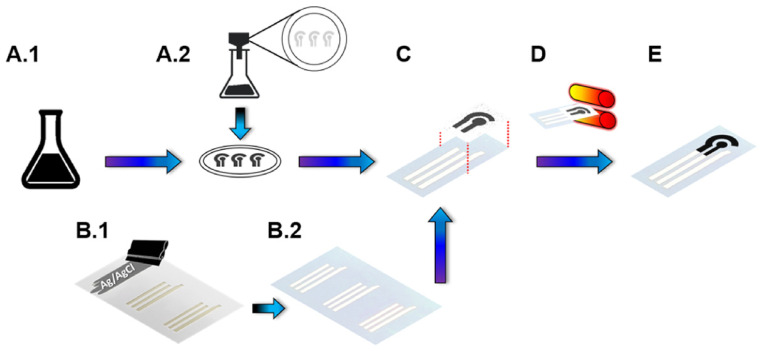
Schematics illustrating the representation of the BH-film-equipped electrode design and assembly. (**A**) Biochar film preparation; (**A.1**) biochar dispersion; (**A.2**) biochar vacuum filtration throughout the stencil filter mask attached to the filter membrane; (**B**) Electrode base preparation; (**B.1**) Ag/AgCl ink-printing of contacts and reference electrode through a stencil printing mask; (**B.2**) printed Ag/AgCl contact after stencil mask peel-off; (**C**) BH-film and electrode base alignment; (**D**) BH-film transfer by thermal lamination (the electrical contacts were insulated with a PET-EVA complementary insulating layer; not shown for clarity); (**E**) Assembled BH-film-equipped electrode. Reprinted with permission from Ref. [32]. Copyright © 2021, American Chemical Society.

Since biochar is catalytic, it can be directly used to make electrodes, such as screen-printed electrodes and carbon-paste electrodes. As stated above, Bukhari et al., used the prepared BH-CNF to directly fabricate an electrochemical sensor [32]. As graphically shown in Figure 2, a BH-CNF-based sensor was constructed following five steps: BH-film preparation, electrode-base preparation, BH-film and electrode-base alignment, BH-film transfer by thermal lamination, and an assembled BH-film-equipped electrode. The procedure for fabricating a sensor is cost-effective and within everyone’s reach. The potential of the BH-CNF-based sensors has been directed toward a wide range of analytes containing phenol moieties and applied in the detection of *o*-diphenols and *m*-phenols in olive oil samples. 

Carbon-paste electrodes are more convenient than screen-printed electrodes in the aspects of preparation. The reason is based not only on the procedure of the preparation, but also on the fact that the demand of the mixed modifier in the preparation of carbon-paste electrodes is easier and lower than that of screen-printed electrodes. Kalinke et al., have carried out several works regarding the preparation of carbon-paste electrodes by mixing biochar as the modifier [33,34]. For example, their recent work involved the preparation of biochar by pyrolysis of a castor cake waste biomass at 400 °C [34]. After activation by HNO_3_ refluxing to increase the amount of oxygenated functional groups, the activated biochar was used to prepare a carbon-paste electrode together with mineral oil and graphite powder. The prepared carbon-paste electrode showed excellent electrochemical responses to a caffeic acid preconcentration, compared to the responses of the electrodes without biochar. Under optimized conditions, the proposed sensor presented a wide linear range, good repeatability and reproducibility for consecutive measurements, high sensitivity (11.06 μA·mM), and low LOD (30.9 nM). It is interesting that the method was successfully applied for caffeic acid detection in real and spiked wine samples without sample pretreatment.

However, to enhance the sensitivity of biochar-based electrochemical sensors and biosensors, the catalytic performance of biochar still needs to be improved. The preparation of functional biochar is an effective method for obtaining biochar with good catalytic properties. The technique for controlling the preparation of biochar modified with various functional groups, nano- or micro-sized biochar, and biochar with various spatial three-dimensional structures is still ongoing by researchers from various fields.

### 2.3. Carrier

Biochar’s porous structure gives it a larger specific surface area for absorbing target analyte and carrying or loading various activate molecules or materials, such as copper hexacyanoferrate [9], Fe_3_O_4_ [35], MoSe_2_ [36], and nickel hydroxide [37]. These materials’ good properties in various aspects of spatial structure, redox ability, magnetism, or conductivity enhance and improve the analytical performance of biochar-based sensors. Summarizing the recent works about metallic nanoparticles/biochar composites, the key effect of biochar is that its porous structure can effectively disperse metallic nanoparticles, and thus make the nano-sized metal provide more activated sites and show enhanced catalytic, redox, or conductive capacities. Therefore, the preparation of metallic nanoparticles (bismuth [38], mercury [39], cobalt [40], and gold [11]) and biochar nanocomposite yields materials with an excellent analytical and electrochemical performance, combining biochar and metal features in the same device [41].

**Figure 3:**
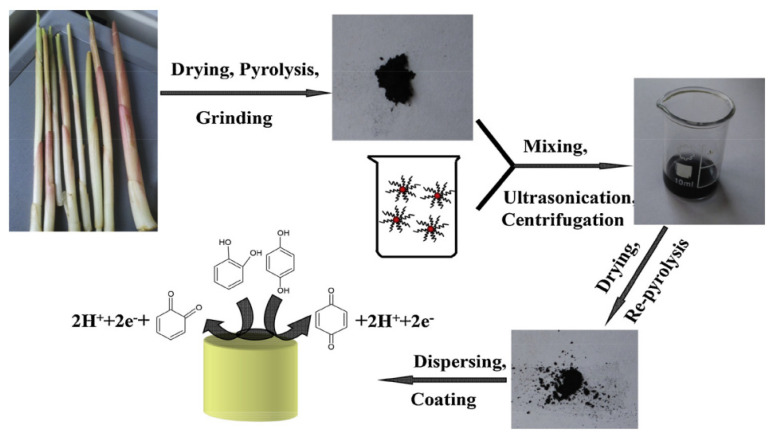
Schematics for the illustration of WBC/Au material preparation and application. Reprinted with permission from Ref. [42]. Copyright © 2020, Elsevier.

For instance, Wang et al., dispersed colloidal Au nanoparticles into a biochar suspension liquid, and then, after a drying process, the mixture was pyrolyzed again. In other words, they prepared the Au nanoparticles and decorated the seedling of white myoga ginger-derived biochar (WBC/Au) by a pyrolysis-dispersion-pyrolysis method. The procedures of the preparation of the biochar and the fabrication of the electrochemical sensor are shown in Figure 3. Due to the dispersion of the Au nanoparticles, the prepared WBC/Au exhibited great electrochemical catalytic properties with respect to hydroquinone and catechol. As a result, a WBC/Au-based electrochemical sensor for the simultaneous determination of hydroquinone and catechol with LOD of 0.002 μM, was successfully developed [42]. The selectivity of a WBC/Au-based electrochemical sensor is excellent, because 500-fold-SO_4_^2−^, K^+^, Na^+^, Cu^2+^, Fe^2+^, Mg^2+^, NO_3_^−^, Cl^−^, NH_4_^+^, 20-fold *p*-chlorophenol and L-cyteine, 50-fold bisphenol A, and 10-fold ascorbic acid, resorcinol, uric acid and glycine did not affect the simultaneous determination of hydroquinone and catechol.

In addition to an inorganic catalyst load, a bio-enzyme can also be loaded on the eco-friendly biochar, because it is very biocompatible with biological molecules and there are many oxygen groups on the surface of biochar, such as carboxylics, ketones, and aldehydes. In the study of electrochemical biosensors, tyrosinase (TYR) enzymes, laccase, horse radish peroxidase (HRP), and glucose oxidase (GOx) are very popular enzymes used for specific detection. Therefore, the oxygen groups of biochar can be used as active sites to anchor biomolecules through a crosslinking reaction. Recent electrochemical biosensors based on biochar involve the immobilization of these enzymes onto biochar.

**Figure 4:**
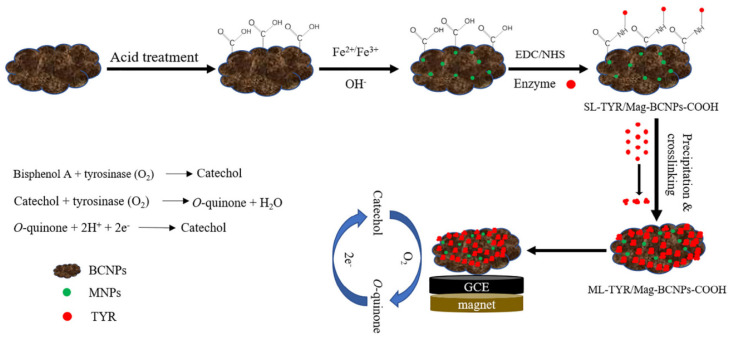
Schematics for the illustration of tyrosinase (TYR) immobilization on Mag-BCNPs-COOH and the electrochemical reaction for the detection of BPA. Reprinted with permission from Ref. [43]. Copyright © 2020, Elsevier.

As shown in Figure 4, He et al., prepared BCNPs from bagasse waste showing great electrochemical activity, and after functionalization with carboxylic functional groups and magnetic nanoparticles (Mag-BCNPs-COOH), the preparation was used for multi-layered TYR immobilization with a covalent attachment, precipitation, and cross-linking method (ML-TYR/Mag-BCNPs-COOH) [43]. Owing to the great properties of the ML-TYR/Mag-BCNPs-COOH, the prepared electrochemical biosensor was used for the detection of bisphenol A (BPA), and an LOD of 2.78 nM with linear ranges from 0.01 to 1.01 μM was obtained.

It is interesting that Naghdi et al., found that using biochar to immobilize laccase could improve the stability and catalytic activity of laccase [44]. The biochar-loaded laccase showed a wider pH tolerance and long-term storage activity, compared with those of a free enzyme of laccase. In addition, after combining the biochar-loaded laccase with iron, the composite obtained even more interesting results. For instance, Zhang et al., immobilized laccase on a cost-effective and nanosized magnetic biochar (L-MBC) by adsorption, precipitation, and crosslinking, and used it for high performance BPA removal [45]. All of these studies state that the biochar-loaded enzyme has good potential for the application in fabricating electrochemical biosensors.

Another type of popular biomolecule modifying the electrode in electrochemical biosensor studies is the antibody or antigen. Martins et al., developed an electrochemical immunosensor based on biochar for the first time. In detail, they immobilized antibodies against Hantavirus Araucaria onto a biochar-modified carbon-paste electrode to fabricate a label-free electrochemical immunosensor for the detection of Hantavirus Araucaria nucleoprotein. Specifically, the covalent bonding between the biochar and the antibodies enhanced the stability of the immobilization and prevented several drawbacks, such as leaching the biomolecules from the device surface [46].

Although biochar shows excellent properties as a carrier and an absorbent, the possible interference produced from the original biomass (e.g., heavy metals) must be considered and given wider and more thorough study.

## 3. The Application of Electrochemical Sensors and Biosensors Based on Biochar

Biochar is stable, highly aromatic, carbon-rich, and eco-friendly. These advantages make it very suitable for the development of electrochemical sensors and biosensors. However, because the study of biochar-based electrochemical sensors (especially biosensors) is still in its infancy, there are not many works reported. The detection processes are mainly focused on three methods for recognizing analytes, which are based on the properties of biochar discussed above, i.e., absorption, electrochemical deposition, and cross-linking. Therefore, the methods of electrode modification by biochar and the properties of biochar will be different according to the different objects to be tested, as shown in Table 1.

### 3.1. Application in Detecting Heavy Metals

The use of biochar for pre-concentration and voltametric determination of inorganic ions and organic species was reported by several research groups all over the world. These methods are based on stripping procedures and have provided significant improvements in the sensitivity and selectivity aspects of sensor performance. These goals were successfully achieved by combining the highly functionalized surface of biochar with a separate spontaneous accumulation step from the electrochemical cell [41]. For example, Oliveira et al. successfully applied biochar in the detection of heavy metals in functional drinks. They mixed biochar with graphite powder and mineral oil to prepare a carbon-paste electrode for the electrochemical detection of Cu^2+^ in beverages. This work described, for first time, the use of biochar as an electrode modifier in combination with differential pulse adsorptive stripping voltammetric (DPAdSV) techniques for preconcentration and determination of Cu^2+^ ions in spirit drink samples. The sensor had a wide detection range of 1.5 to 31 μM and a low LOD of 0.4 μM for the detection of Cu^2+^. The selectivity of the sensor was also evaluated. The general interference ions did not affect the detection of Cu^2+^ by the proposed electrochemical sensor, such as Fe^3+^ (2.0 mg·L^−1^), Zn^2+^ (3.0 mg·L^−1^), As^3+^ (100 μg·L^−1^), and Pb^2+^ (200 μg·L^−1^) [52]. 

Generally, the stripping voltammetry is used for the determination of metal ions. Other than that, although biochar exhibits excellent absorption properties with heavy metals, the selectivity of the absorption is still a great challenge in the study of biochar-based electrochemical sensors and biosensors. In order to overcome the influence of interference ions, the standard addition method is adopted. For instance, Oliveira et al., reported a carbon-paste electrode based on biochar pyrolyzed from coffee grounds for the detection of lead ions (Pb^2+^) in gunshot residues [59]. The electroanalytical strategy was also based on DPADSV performing the pre-concentration step under an open circuit potential condition separately from the electrochemical cell. In the evaluation of the selectivity of the electrochemical sensor, species such as Sb^3+^, Cu^2+^, Cr^3+^, and Fe^2+^ have been chosen, since these are the main components of gunpowder. Each of the tested metal ions were added to the pre-concentration solution jointly with Pb^2+^, in the fixed concentration of 10 µM, varying from 0.10, 1.0, and 10-fold higher than Pb^2+^. The results showed that all metals tested caused significant interference in the Pb^2+^ determination signal for lower, equal, and higher concentrations. Since carbonaceous elements have a high capacity for interacting with different inorganic compounds, it was possible to relate the changes in the voltametric signals with the concomitant species’ presence. Therefore, these metals’ presence may have caused inhibition of the accumulation of Pb^2+^ on the electrode surface in the analyte’s pre-concentration step, with a probable competition between the species and the Pb^2+^ occupying the active sites present in the biochar. Due to this, an evident decline in the response signal of the lead ions was observed. To overcome this effect, the standard addition method was chosen to quantify Pb^2+^ in real samples.

**Figure 5:**
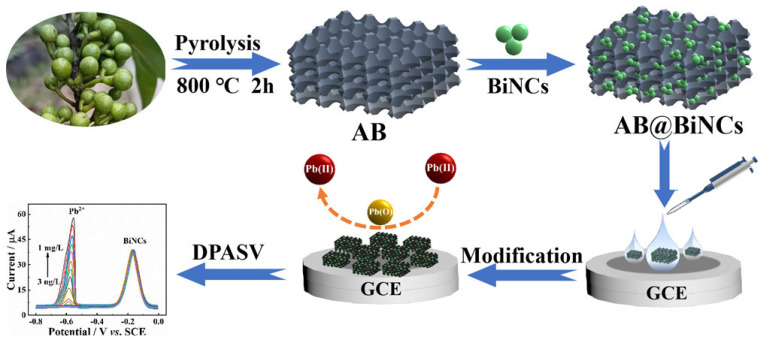
Schematics for the illustration of the fabrication process of the BiNCs@AB composite sensor for the sensing of Pb^2+^. Reprinted with permission from Ref. [38]. Copyright © 2021, American Chemical Society.

Another work concerning the detection of Pb^2+^ was reported by Zou et al. [38] Considering the low electrical conductivity and easy agglomeration of Bi particles, porous activated biochar (AB) with the merits of high electrical conductivity, large surface area, and good chemical stability was chosen as the support material. In summary, a ratiometric electrochemical platform based on the BiNCs@AB-modified electrode was constructed for the analysis of Pb^2+^ in a linear range from 3.0 ng·L^−1^ to 1.0 mg·L^−1^ with an LOD of 1.0 ng·L^−1^. As shown in Figure 5, for the design of the sensing material, hierarchical porous AB was employed as the substrate material for the in situ growth of BiNCs. The prepared BiNCs@AB composite possesses a large active area, fast electron transfer ability, high mass transfer efficiency, and a strong enrichment capacity for Pb^2+^. Additionally, the oxidation signal of BiNCs served as the internal reference, which greatly raised the reproducibility and reliability of the sensor. Benefiting from the synergy of BiNCs and AB, the proposed sensor exhibited excellent sensitivity, good selectivity (the influence of 50 fold concentrations of Hg^2+^, Na^+^, Zn^2+^, NO_3_^−^, K^+^, Cl^−^, Al^3+^, Cu^2+^, SO_4_^2−^, Co^2+^, and Cd^2+^ were tested), and high stability in the detection of Pb^2+^, and was successfully applied in monitoring Pb^2+^ in real water samples. 

In addition to the detection of single heavy metal ions, the simultaneous detection of two heavy metal ions was achieve based on the different potential of the two heavy metal ions on the sensing surface of a biochar-modified electrode. Recently, Ademar et al., reported a highly robust sensor that exhibited an excellent analytical response in the simultaneous quantification of trace Cd and Pb ions. The method was developed via modifying glassy carbon electrodes with biochar, nanodiamonds, and chitosan. It is interesting that the sensing process had no need for step cleaning of the electrode surface to remove any remaining bound metals. In addition, the analysis of the Cd and Pb ions indicated a high electrochemical sensitivity of 0.42 and 5.30 μA·μmol^−1^·cm^−2^ in the quantification of cadmium and lead, respectively [62].

### 3.2. Application in Detecting Pesticide and Veterinary Drug Residues

Pesticide and veterinary drug residues are generally organic molecules, or their mixture. The procedure of detection always involves absorption and a following electrochemical redox reaction, which is the same in the detection of heavy metals. The difference in detecting heavy metals by means of the electrochemical analysis method is that the detection of pesticide and veterinary drug residues is always accomplished via the differential pulse voltammetry (DPV) method. 

Zhang et al., reported an electrochemical sensor for the portable intelligent detection of clenbuterol (CLB) [51]. In their work, Kudzu, a traditional Chinese medicine, was pyrolyzed to produce biochar, which was used as the modified material of the flexible electrode. At the same time, the wireless intelligent sensor detection of CLB was realized by using a micro-analyzer composed of a smart phone and a micro electrochemical workstation. The smart portable electrochemical sensor had the advantages of simple preparation, small volume, high stability, good repeatability, and low cost. The sensor had a wide detection range of 0.95–14.31 μM and a low LOD of 0.75 μM for CLB detection. In addition, the results showed that a wireless intelligent portable sensor based on a micro-analyzer has the potential to realize the field detection of CLB. 

As notable as CLB in the field of veterinary drug residues, ractopamine (RAC) as an important *β*-agonist that is frequently found in pork to enhance food quality in foods that are harmful to human health [63]. Cao et al., developed a type of electrochemical biosensor using a biochar-supported Cu^2+^/Cu^+^ composite as an electrochemical sensing interface for detecting RAC [28]. The electrochemical sensor combined the collective advantages of Nafion (enriching RAC and effectively shielding the interference of negatively charged compounds), biochar (a unique three-dimensional porous network structure to increase the contact area), and Cu^2+^/Cu^+^ (with excellent electrical conductivity to speed up the charge transfer rate), which are beneficial in the accumulation and electrochemical sensing of targets on a Nafion-biochar-supported Cu^2+^/Cu^+^ electrode (NBC-GCE). The sensor, as prepared, showed a good electrochemical sensing ability, with an ultralow LOD and high sensitivity (0.041 μM and 416 μA·mM^−1^·cm^−2^, respectively), in the range of 0.1 to 1.75 μM. In addition, the stability, repeatability, and anti-interference performance of the modified electrode were also satisfying. Finally, its practicability was shown for assaying RAC in pork sausages.

### 3.3. Application in Detecting Environmental Estrogen

**Figure 6:**
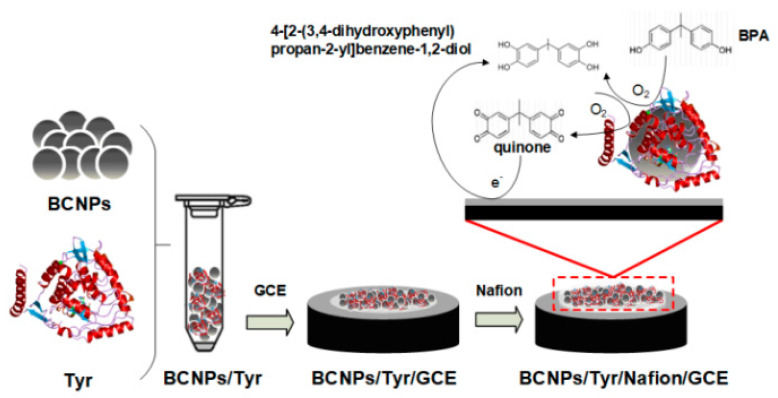
Schematics for the illustration of the procedures for the preparation of the BCNPs/Tyr/Nafion/GCE biosensor and the principle of electrochemical BPA sensing. Reprinted with permission from Ref. [49].

Bisphenol A (2, 2-bis(4-hydroxyphenyl) propane, BPA) is a phenolic compound and an endocrine disrupting compound that shows estrogenic activity, and may have adverse effects on humans, wildlife, and reproductive systems [64]. A series of electrochemical biosensors based on porous carbon-based composite materials were developed for the detection of phenols, as shown in Table 1. Among these reported electrochemical sensors for the detection of BPA, the enzyme biosensor must be mentioned; its detection is different from that of the other organic molecules. Tyrosinase as an ortho-hydroxylation oxidase was used as the signaling enzyme because it possesses catalytic bioactivity for BPA that can be oxidized sequentially to polyhydric phenol and the electrochemically active o-quinone, where the o-quinone can be further reduced at the electrode surface, producing a reducing current signal proportional to the concentrations of BPA [65]. Based on this, Liu et al., reported a tyrosinase enzyme biosensor using a highly conductive sugarcane-derived biochar nanoparticle (BCNP) as a transducer and signal enhancer (BCNPs/Tyr/Nafion/GCE) for the sensitive detection of BPA [49]. The fabrication procedure and the detection mechanism of the BPA sensor is schematically shown in Figure 6. This might be the first time that biochar had been proposed for use in conjunction with enzymes in the biosensor. The prepared biochar nanoparticles had large specific surface areas, good conductivity, biocompatibility, and environmental friendliness, which were beneficial in enhancing the activity of the enzyme. Under the optimized conditions, it could detect BPA with good sensitivity, with a linear range from 0.02 to 10 µM, and an LOD of 3.18 nM. The selectivity of the biosensor toward 3 μM BPA in the presence of various ionic interferents (with 100-fold concentration higher than BPA), such as Cl^−^, Ca^2+^, SO_4_^2−^, and Mg^2+^, was assessed and the detection of BPA was not affected.

In another recent work reported by He et al., they immobilized tyrosinase enzymes onto highly conductive magnetic biochar nanoparticles (BCNPs) with carboxyl functionality (Mag-BCNPs-COOH) by covalent, cross-linking, and precipitation methods to fabricate a sensitive and reusable electrochemical biosensor for the detection of BPA [43]. It was found that the composite has good electrochemical activity, and the multilayer fixed composite (ML-TYR/Mag-BCNPs-COOH) had a much higher current response to BPA than did a single layer (SL-TYR/Mag-BCNPs-COOH). Under optimized conditions, the developed electrochemical sensor could detect BPA in a linear range between 0.01 to 1.01 μM, with a low LOD of 2.78 nM. 

Catechol (CA) and hydroquinone (HQ) are also harmful to environmental water. To sensitively determine them, Liu et al., prepared N-doped porous biochar (NPB) and then developed an electrochemical sensor for the simultaneous detection of CA and HQ [50]. They considered that the mechanism for realizing the simultaneous detection of CA and HQ by NPB is attributable to three aspects: (1) The 3D pore structures of NPB could provide effective channels for electron transfer; (2) the mesopores/macropores could increase the effective surface area and allow easy access of the electrode/electrolyte interface, resulting in effective electrocatalytic behaviors for HQ and CA; and (3) The N doping facilitates the adsorption of H- and H-H bonds. Therefore, a small amount of N doping could improve the enrichment capacity of NPB for the targets HQ and CA [50].

Tetrabromobisphenol A (TBBPA) is a kind of brominated flame retardant, which can be released into the environment and could seriously threaten human health [66]. Luo et al., developed a simple and cost-effective electrochemical sensor based on an Fe_3_O_4_-activated biochar. The proposed sensor showed excellent electrochemical performance toward TBBPA-sensing when compared to separate precursor materials [35]. The electrochemical test results revealed that the Fe_3_O_4_-activated biochar film exhibited a lower charged transfer resistance, a larger active surface area, and a higher accumulation efficiency toward TBBPA. Such merits enabled the prepared electrochemical sensor to achieve good performance for the detection of TBBPA in the linear range between 5 to 1000 nM, with a low LOD of 3.2 nM.

### 3.4. Application in Detecting Organic Pollutants

**Figure 7:**
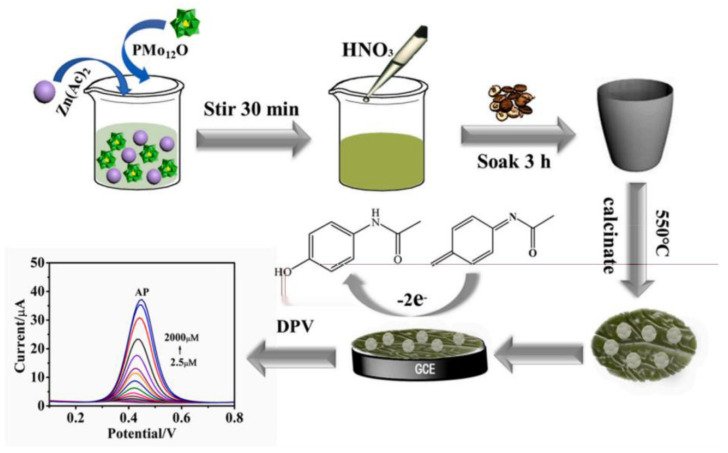
Schematics for the illustration of the reparation of ZnO-MoO_3_-C composites and the detection procedure of the MIP electrochemical sensor. Reprinted with permission from Ref. [52]. Copyright © 2021, Elsevier.

According to the physicochemical properties of organic pollutants, the molecular imprinted polymers (MIPs)-based electrochemical sensing technique is considered to be one of most effective electrochemical assays in the detection of organic pollutants [67]. In the biochar-based electrochemical sensors, sensing organic pollutants are not exceptions. As shown in Figure 7, Liu et al., prepared a ZnO-MoO_3_-biochar nanocomposite with a 2D structure by pyrolyzing mushroom-derived carbon nanosheets and Zn_1.5_PMo_12_O_40_ polyoxometallate [52]. Following that, the ZnO-MoO_3_-biochar nanocomposite was used to modify the GCE and thereafter used as a stable base film for the formation of MIPs. By means of the DPV technique, the developed MIP electrochemical sensor exhibited selectively and sensitively for AP with a wider linear dependence on the concentration of Acetaminophen (AP), from 2.5 to 2000 μM, and a lower LOD of 1.14 μM, compared with other carbon-base-material-modified GCE and GCE electrodes.

In addition to the formation of MIPs on the modified electrode by dipping it into the polymer reaction solvent, the electrochemical deposition of MIPs on the modified electrode is also a frequently used method for the fabrication of MIP electrochemical sensors. Zhou et al., prepared a corncob biochar layer that supported a chitosan-MIP electrochemical sensor for the determination of dibutyl phthalate (DBP) [53]. In the fabrication process, after the functional corncob biochar (F-CC_3_) was cast on the GCE to form a F-CC_3_ film, the MIP film was obtained through an electrochemical deposition method with chitosan as the functional monomer, glutaraldehyde as the crosslinking agent, and DBP as the template molecule. This developed sensor took excellent advantage of the MIPs technique and the F-CC_3_ biochar to achieve high selectivity (toward dimethyl phthalate and butyl benzyl phthalate) and high sensitivity in the detection of DBP in a linear range of 0 to 1.8 μM with an LOD of 2.6 nM [53].

### 3.5. Application in Biosensors

As stated above, the eco-friendly and biocompatible characteristics of biochar make it very suitable for the fabrication of electrochemical biosensors. Ayten et al., prepared Fe_3_O_4_-biochar (Fe-BC) nanocomposites by using hazelnut-shell (HNS) biochar and FeCl_2_ in several different methods [54]. Then, they used Fe-BC nanocomposites to develop an electrochemical biosensor for the detection of H_2_O_2_. Among the prepared biochar by means of the different prepared methods, the iron-containing biochar prepared by acid and microwave activation showed the best electrocatalytic performance in H_2_O_2_ reduction [54]. After optimization, the electrochemical biosensor found that the detection of H_2_O_2_ is linear in the range of 0.5–10 mM. The interference effects of L-cystine, ascorbic acid and urea in the detection of H_2_O_2_, along with the detection of H_2_O_2_ in milk samples, were also studied and Fe-BC via microwave-assisted pyrolysis showed excellent selectivity.

Using biochar’s carrier and catalytic capacities, Kalinke et al., fabricated a non-enzymatic electrochemical biosensor based on nickel oxyhydroxide supported by activated biochar in the detection of glucose in human saliva and blood serum [37]. Moreover, the sensing platform was assembled as a microfluidic device, which was a plastic platform that was printed using a 3D printer. The prepared non-enzymatic amperometric glucose biosensor, coupled with μTED, showed a good repeatability of 3.84% for successive injections of glucose (*n* = 10) and a linear dynamic range from 5.0 to 100.0 µM with an LOD of 0.137 µM and a limit of quantification (LOQ) of 0.457 µM in the detection of glucose.

**Figure 8:**
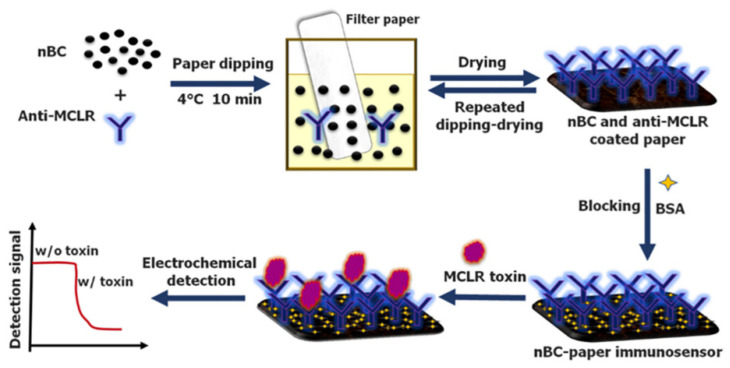
Schematics for the illustration of the procedures for construction of nBC-paper immunosensor and the electrochemical detection of MCLR. Reprinted with permission from Ref. [56]. Copyright © 2021, Elsevier.

Biochar can not only carry enzyme, but also other biomolecules, such as antibodies. However, there have been few reports about biochar-based electrochemical immunosensors until now. More recently, Yao et al., reported a portable, cheap, and sensitive paper type of electrochemical immunosensor that was fabricated with conductive nano-sized biochar paper, as the conductive layer for the sensitive detection of microcystin-LR (MCLR) toxin in water. As shown in Figure 8, the highly conductive nanosized biochar particles were successfully utilized for the cost-effective and reproducible paper-type electrochemical immunosensor (nBC-paper immunosensor) construction. In the optimized conditions, the electrochemical immunosensor showed a wide linear range of 0.1–100 nM, with a low LOD of 17 pM, in the determination of MCLR. Moreover, the proposed nBC-paper immunosensor could be successfully employed for the detection of the real environmental water [56].

### 3.6. Applications in Detecting Other Subjects

Due to the excellent properties of biochar, it has been also used to fabricate electrochemical sensors for the detection of other subjects. Rao et al., developed a one-step green preparation of kudzu vine biochar decorated with graphene-like molybdenum selenide (MoSe_2_), with oxidase-like activity as an intelligent nanozyme-sensing platform for the voltametric detection of hesperetin (HP) in orange peel, using the in situ hydrothermal synthesis method. The electrochemical detection mechanism of HP was such that the reversible electrochemical oxidation of HP with the hydroxy group in the ring B led to the formation of quinone, with a 2-electron and 2-proton process. They also proposed an emerging machine learning (ML) technique based on artificial neural network (ANN) algorithms for the intelligent analysis of sensing [36].

The simultaneous detection of two different subjects is always a controversial research point because high efficiency and high output are highly demanded in the fields of various sample analysis. Ferreira et al., proposes a pure biochar from a palm residue as an electrode modifier of GCE for the simultaneous detection of organic compounds that present similar peak potentials [10]. Because biochar is modified on the GCE, the sensitivity, electrocatalytic activity, and peak separation of molecules with close or overlapped peak potentials were improved. As a proof of concept, three pairs of molecules were successfully evaluated: catechol (CC) and hydroquinone (HQ); levofloxacin (LEVO) and norfloxacin (NOR); and tert-butylhydroquinone (TBHQ) and butylated hydroxy-anisole (BHA). In this sense, biochar is a low-cost, easy-to-prepare, and a promising for use in the simultaneous analysis of similar organic compounds [10].

**Figure 9:**
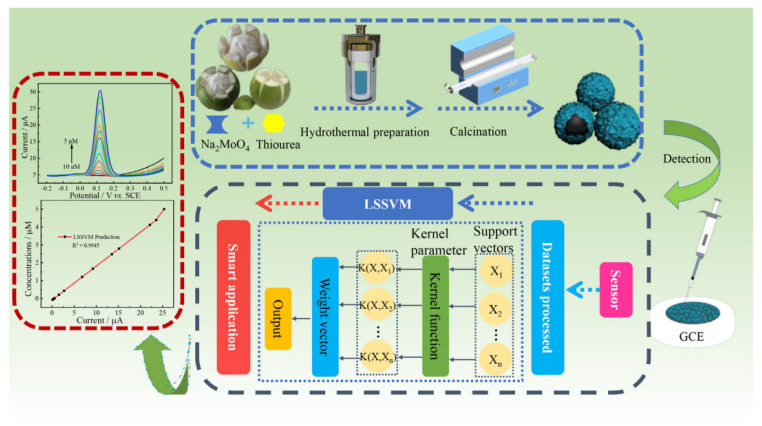
Schematics for the illustration of the preparation of the a-MoS_x_-BM nanocomposite for smart sensing baicalin. Reprinted with permission from Ref. [59]. Copyright © 2021, Elsevier.

Recently, Rao et al., reported a least squares support vector machine (LSSVM) [59]. It consists of an optimized algorithm based on a support vector machine (SVM), compared with an artificial neural network (ANN) that applies a set of linear equations instead of the quadratic programming formula in the standard SVM. As shown in Figure 9, they prepared a MoS_x_-biochar microsphere (BM) nanocomposite by a green and efficient hydrothermal method, and the discarded inedible pomelo peel was selected as the precursor of BM. MoS_x_-BM was employed as the voltametric sensing platform for baicalin, due to the synergistic effect of both BM (good conductivity) and MoS_x_ (both environmental stability and electrocatalytic capacity). The nanocomposite displayed excellent sensing performance in the linear range, between 10 nM and 5 μM with low LOD of 2 nM for the determination of baicalin [59].

## 4. Challenges and Prospects for the Study of Electrochemical Sensors and Biosensors Based on Biochar

For environmental protection, sustainable development, and the achievement of carbon peaking and carbon neutrality goals, there are many merits in applying biochar in various fields, as well as with electrochemical sensors and biosensors; for example, low-cost, easily obtained, green preparations and the reuse of agricultural waste. Nevertheless, regarding the electrochemical sensing technique, there are still key challenges and issues to be addressed for the application of biochar in the fabrication of electrochemical sensors and biosensors, with their inherent demands of precision and repeatability. Additionally, the efficiency and the catalytic and absorbent properties of biochar still need to be improved to enhance the sensitivity and stability of electrochemical sensors and biosensors in either the development of theoretical models of measurement or the improvement of the sensor construction process.

(1) From the above works and other reported works, the mesopores/macropores structure of biochar is very beneficial for sensing surface construction and can provide biochar with excellent properties. Generally, the surface area and pore size of biochar increases with rising pyrolysis temperatures [68]. However, higher temperatures could result in the porous structure being destroyed and blocked [69] and, more importantly, higher pyrolysis temperatures would decrease the number of functional groups [70]. Therefore, how to improve the biochar sorption capacity and thus improve the electrochemical response signal need to be addressed.
(2) It is worth noting that various redox couples of metal ions have been used as the redox probe in the fabrication of electrodes. Although the metal ions exhibit excellent redox properties, the dispersibility of metal ions in biochar still need to be improved because of the great influence of the redox property in dispersibility. Therefore, how to carry, absorb, or wrap redox metal ions and thus improve the dispersibility of metal ions in the biochar need to be considered further.
(3) The application of biochar is increasingly popular because one of its advantages is low-cost. However, the current technique of preparing biochar always needs a high-temperature treatment. Therefore, it is still a significant challenge to optimize the synthesis process for a mild and low-energy-consuming condition. Additionally, further studies are needed to prepare activated biochar that has a special structure and abundant functional groups under low temperatures.
(4) Whether used as an absorbent, a catalyst, or a carrier, the possible interference produced from the original biomass (e.g., from heavy metals) must be considered, because such interference might contaminate the analytes or affect the repeatability of the sensors, which is an important technical indicator for the evaluation of sensors and biosensors [71,72]. Therefore, how to realize the controlling preparation of biochar with uniform size and composition still needs wider and more thorough study.

## 5. Conclusions

In brief, biochar has received great attention since its production. By virtue of its stable, highly aromatic, carbon-rich and eco-friendly advantages, biochar is a good candidate material for electrode fabrication or modification, and it has played important roles in preparing electrochemical sensors and biosensors, such as being as carrier, absorbent, and catalyst. Because the study of biochar-based electrochemical sensors (especially biosensors) is still in its infancy, there are not many works reported, and the detection targets are focused on uncommon types of subjects in environmental pollutants and food contaminants. Furthermore, studies on the methods of electrode modification by biochar and the properties of used biochar that will be different, according to the different objects to be tested, are still ongoing. In short, we think that with the development of precise control preparation of biochar, biochar-based electrochemical sensors and biosensors receive increasing attention and broader applications.

## Figures and Tables

**Table 1:** Recent reports about the application of biochar in the fabrication of electrochemical chemical sensors and biosensors.

Target Analyte	Biomass Resource	Preparation Method	Types of Biochar for Electrode Modification	Analysis Technique	Linear Range (μM)	LOD (nM)	Real Sample	Ref.
17*β*-estradiol	Bagasse	Pyrolysis	Biochar	DPV	0.05–20	11.30	Ground water	[27]
Acetaminophen	Mushroom	Pyrolysis	Zno-moo3/biochar	DPV	2.5–2000	1140	Blood serum and Tablet	[47]
Baicalin	Pomelo peel	Hydrothermal synthesis & Pyrolysis	A-mosx/biochar	DPV	0.01–5	2	Shuang-Huang-Lian oral liquid	[48]
Bisphenol A	Bagasse	Pyrolysis	Tyrosinase/biochar	I-t	0.02–10	3.18	Ground water	[49]
Bisphenol A	Bagasse	Pyrolysis	Magnetic nanoscale biochar/tyrosinase	I-t	0.01–1.01	2.78	Environmental water	[43]
Carbendazim	Eichhornia crassipes	Pyrolysis	Reduced graphene oxide/biochar	DPV	0.03–0.9	2.3	Orange juice, lettuce leaves, drinking water, and wastewater	[41]
Catechol and hydroquinone Simultaneously	Rice flour; Urea; Sodium citrate	Hydrothermal synthesis	N-doped porous biochar	DPV	0.4–15 and 0.4–20	37 and 47	-	[50]
Catechol and hydroquinone; levofloxacin and norfloxacin; tert-butylhydroquinone and butylated hydroxy-anisole.	Babassu petiole	Pyrolysis	Ferrocyanide/biochar	SWV	-	-	-	[10]
Clenbuterol	Kudzu vine	Pyrolysis	Zn/biochar	DPV	0.95–14.31	750	Bovine serum	[51]
Cu^2+^	Castor oil cake	Pyrolysis	Biochar	DPASV	1.5– 31	400	Spirit drinks	[52]
Dibutyl phthalate	Corncob	Pyrolysis	MIP/biochar	DPV	0–1.8	2.6	Rice wine	[53]
Glucose	Eggshell membrane	Pyrolysis,	Cu^2+^–Cu^+^/biochar	I-t	12.5–70	1040	Human serum	[29]
Glucose	Castor oil cake	Pyrolysis	Ni/biochar	I-t	5.0–100.0	137	Human saliva and blood serum	[37]
Glucose	Waste microalgal sludge	Pyrolysis	Co/chitosan/biochar	I-t	-	-	-	[40]
Glyphosate herbicide	Babassu petiole	Pyrolysis	Copper hexadecafuoro-29H/biochar	SWV	0.3–4	20	Lake water and River water	[9]
H_2_O_2_	Hazelnut shell	Microwave assisted pyrolysis	Fe_3_O_4_/biochar	I-t	600–10,000	503 × 10^3^	Milk	[54]
Hantavirus nucleoprotein	Castor bean	Pyrolysis	Biochar	CV	5.0 ng·mL^−1^–1.0 μg·mL^−1^	0.14 ng·mL^−1^	Human serum	[46]
Hesperetin	Kudzu vine	Hydrothermal synthesis	MoSe_2_/biochar	DPV	0.01–9.5	2	Oranges	[36]
Hydroquinone Catechol	White myoga ginger	Pyrolysis	Au/biochar	DPV	0.008–1.0 and 1.0–7.0; 0.01–1.0 and 1.0–7.0	2; 4	Tap water	[42]
Hydroquinone Catechol	Dracaena sanderiana	Co-pyrolysis	Au/biochar	DPV	0.01–0.2 and 0.2–10; 0.04–0.4 and 0.4–15	3.4; 9.0	Local tap water	[11]
Isoniazid	Castor oil cake	Pyrolysis	Copper hexacyanoferrate/biochar	I-t	1.0–10	63	Human urine	[55]
Methyl parathion	Castor oil cake	Pyrolysis	Biochar	DPV	0.1–70	39	Tap water	[31]
Microcystin-LR	Sugarcane waste	Pyrolysis	Antibody/biochar	I-t	0.1 × 10^−3^–0.1	0.017	Lake water and River water	[56]
NH_3_(g)	Corn stover	Pyrolysis & Solvent casting method	Polylactic acid/biochar	LSV	80–170 ppm	80 ppm		[57]
Paraquat	Water hyacinth	Pyrolysis	Rgo/biochar	DPV	0.74–9.82	20	Coconut water, wastewater, honey, lettuce and lemon	[58]
Pb^2+^	Spent coffee grounds	Pyrolysis	Biochar	DPASV	0.128–2.44	4.5	Gunshot residues and hair dye	[59]
Pb^2+^	Peach wood	Pyrolysis,	Biochar	SWASV	0.5–120 μg·L^−1^	0.02 μg·L^−1^	Tap water	[60]
Pb^2+^	Litsea cubeba shell	Pyrolysis & solvothermal method	Bismuth nanocluster/biochar	DPASV	0.014 × 10^−3^–4.83	0.005	Paddy water	[38]
Pb^2+^; Hg^2+^	Magnolia grandiflora fruit	Pyrolysis	Biochar/uio-66-NH_2_/biochar	DPASV	0.001–1000 μg·L^−1^	0.3 ng·L^−1^	Lake water and paddy water	[61]
Pb^2+^; Cd^2+^	Babassu petiole	Pyrolysis	Nanodiamonds/Biochar/Chitosan	SWASV	1.0–75.0; 0.25 –6.00	110; 56	River water	[62]
RAC	Eggshell membrane	Pyrolysis,	Cu^2+^–Cu^+^/biochar	DPV	0.1–1.75	41	The pork sausage	[28]
Tetrabromo-bisphenol A	Excess sludge	Pyrolysis	Fe_3_O_4/_biochar	DPV	0.005–1	3.2	River water	[35]

## Data Availability

Not applicable.
